# 283 Beyond Utilization: Measuring Antimicrobial-Associated Adverse Drug Events in Hospitalized Children

**DOI:** 10.1017/ash.2026.10644

**Published:** 2026-06-23

**Authors:** Lisa Hall, Josephine Wen, Michael J Malloy, Karin Thursky, Rodney James, Noleen Bennett

**Affiliations:** 1 The University of Queensland; 2 Royal Melbourne Hospital Guidance Group; 3 Melbourne Health; 4 University of Melbourne; 5 National Centre for Antimicrobial Stewardship; 6 Peter Doherty Institute for Infection and Immunity

## Abstract

**Background:** There is increasing awareness of the importance of antimicrobial stewardship in long-term care settings internationally. Despite this, there is little robust surveillance data on the quality of prescribing in this setting to help target interventions. In Australia, nursing homes are known as residential aged care homes (RACHs). The Aged Care National Antimicrobial Prescribing Survey (Aged Care NAPS) is a national, standardized audit program enabling Australian RACHs to monitor antimicrobial use and provide feedback on prescribing practices. Since 2016, the program has collected detailed prescribing data but has not made an explicit assessment on prescribing quality. With revision to Australian national prescribing guidelines, we now have the potential to use this data to assess concordance with guidelines. **Methods:** All Australian RACHs are eligible and invited annually to participate in the Aged Care NAPS. A single-day point prevalence audit is the primary methodology adopted. Antimicrobial prescribing data are collected by auditors at participating RACHs and entered into an online platform. All residents present on the survey day were included in the audit. Retrospective analysis of Aged Care NAPS data collected between 1 January 2020 and 31 December 2024 was undertaken. Guideline concordance was evaluated for the five most common indications (cystitis, tinea, non-surgical wound infection, pneumonia and cellulitis), using the Therapeutic Guidelines: Antibiotic 16th edition and Dermatology 4th and 5th editions. **Results:** A total of 41,786 prescriptions from 1,408 RACHs were audited during the study period. The five most common indications accounted for 37.4% of all prescriptions. Incorrect antimicrobial choices were common, such as cefalexin and roxithromycin for pneumonia, and amoxicillin-clavulanic acid and doxycycline for cellulitis. Prevalence of dosing errors ranged from 3.4% (trimethoprim for cystitis) to 78.0% (amoxicillin for pneumonia). Cefalexin was frequently prescribed and commonly incorrectly dosed (70.3% in cystitis, 72.8% in non-surgical wound infections, 68.2% in cellulitis). Over 40% of prescriptions for the top five antimicrobials for each indication exceeded recommended treatment durations, ranging from 41.7% (amoxicillin for cystitis) to 96.8% (ketoconazole for tinea). Prescribing for tinea often exceeded six months and included non-recommended pro re nata (PRN) prescriptions. **Conclusion:** The Aged Care NAPS shows promise as a tool to monitor the quality of antimicrobial prescribing in long-term care facilities. This analysis has identified targets for improving antimicrobial prescribing, focusing on choice, dosing and duration.

